# Tracheal Chondrosarcoma: Systematic Review of Tumor Characteristics, Diagnosis, and Treatment Outcomes with Case Report

**DOI:** 10.1155/2017/4524910

**Published:** 2017-05-23

**Authors:** Emily A. Kutzner, Joshua S. Park, Salman Zaheer, Jared C. Inman

**Affiliations:** ^1^School of Medicine, Loma Linda University, 11175 Campus St., Loma Linda, CA 92350, USA; ^2^Department of Otolaryngology-Head and Neck Surgery, Loma Linda University Medical Center, 11234 Anderson St., Suite 2586A, Loma Linda, CA 92354, USA; ^3^Department of Cardiothoracic Surgery, Loma Linda University Medical Center, 11234 Anderson St., Suite 1637, Loma Linda, CA 92354, USA

## Abstract

To our knowledge this is the first systematic review of tracheal chondrosarcoma treatment outcomes. Management insights are thoroughly discussed. Men constitute 93.8% of cases, and most of these occur in the distal trachea. The most common symptom, dyspnea, occurs in virtually all patients. Extratracheal extension had occurred in 78.6% of patients. Definitive treatment with tracheal resection showed no recurrences in 10 patients with mean follow-up of 3.1 years. Adjuvant radiotherapy may be utilized for improving local control when open complete resection cannot be performed, but only after endoscopic excision of gross tumor.

## 1. Introduction

Tracheal chondrosarcomas are exceptionally rare, with only 17 cases having been reported in the English literature, to date. Moreover, the whole body of literature for different subsites of chondrosarcoma is also underwhelming in its discussion of advanced treatment decisions in high risk or surgically difficult areas to address, specifically multimodality treatment options. A systematic review of the current English-language literature was performed in an effort to further characterize these rare tumors and to determine treatment outcomes with different management techniques—namely, surgical excision with negative margins. Additionally, we include our own case of a 61-year-old male patient with tracheal chondrosarcoma treated with open tracheal resection and end-to-end anastomosis, without adjuvant therapy.

## 2. Materials and Methods

A systematic literature review of all cases of tracheal chondrosarcoma reported in English literature was performed independently by two authors (E.K., J.I.). The PubMed database, Cochrane Library, Web of Science, and Google Scholar were searched, using the medical subject heading terms of “tracheal chondrosarcoma,” “trachea chondrosarcoma”, “chondrosarcoma”, “cartilaginous tumor trachea”, “trachea”, and “tracheal” to accumulate cases reported during the time period of 1966 (when PubMed was officially started) to January 4, 2016. We limited the search to include only cases published in the English-language literature. Inclusion criteria required a diagnosis of primary tracheal chondrosarcoma. The references included in these cases as well as those included in additional relevant articles were incorporated as references in this review. Two additional cases, one with an English abstract listed on PubMed and one referenced in several other case reports, were published in Japanese and were excluded from this study [1, 2]. Another two case reports published in English in 1954 and 1959 (prior to our the start of our inclusion period) were referenced in several of the case reports in this study; however the tumors in these reports were poorly described and relatively uncharacterized [3, 4]. Two authors independently performed the literature search and data extraction, combined their findings, and removed duplicates (E.K., J.I.). Details of the literature selection strategy are diagramed in Figure 2 following the PRISMA statement format from 2009 [5]. Patient data from the case reports were recorded in Excel ©2013 and pertinent, or predominately present, data were analyzed and used for cohort analysis. Each discrepancy or uncertainty was explored and resolved by author consensus. Institutional review board was applied for and granted exemption.

## 3. Results

The description of our experience with a unique case of tracheal chondrosarcoma is detailed below, and our specific patient details are outlined in Table 1, along with the data extracted from the previously reported cases of tracheal chondrosarcoma [6–20]. Our review yielded 15 previously reported cases of tracheal chondrosarcoma in the English literature, the majority of which involved elderly men, with most lesions occurring in the lower trachea. Further data extraction is compiled in Tables 2 and 3 [6–20].

A 61-year-old morbidly obese male patient with a 4-year history of stable obstructive sleep apnea (OSA) presented to Loma Linda University Medical Center Emergency Department with a 2-day history of acute dyspnea. The patient had been experiencing progressively worsening dyspnea over the course of approximately 2 months.

**Figure 1:**
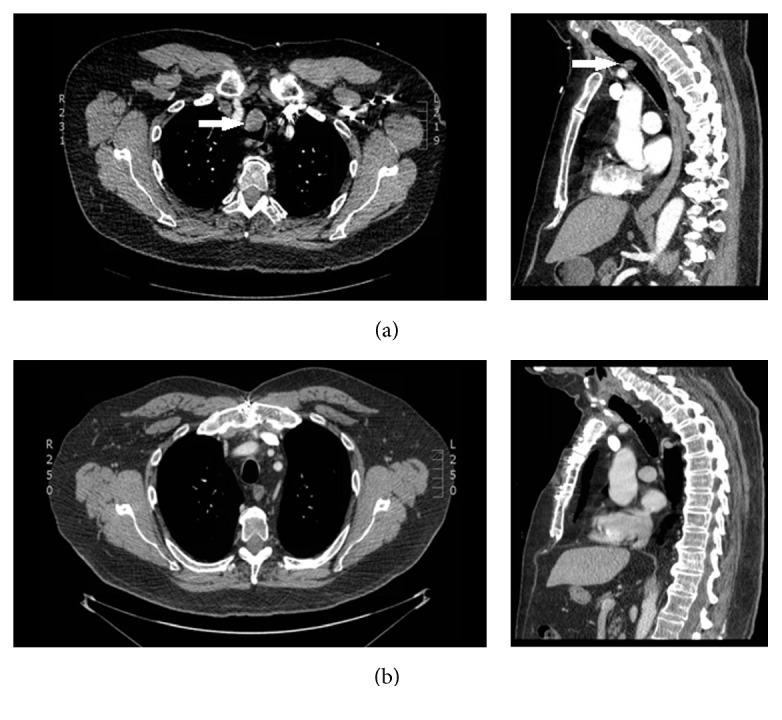
CT scan preoperative axial and sagittal views of the tumor (white arrows, (a)); postoperative views of resected trachea (b).

**Figure 2:**
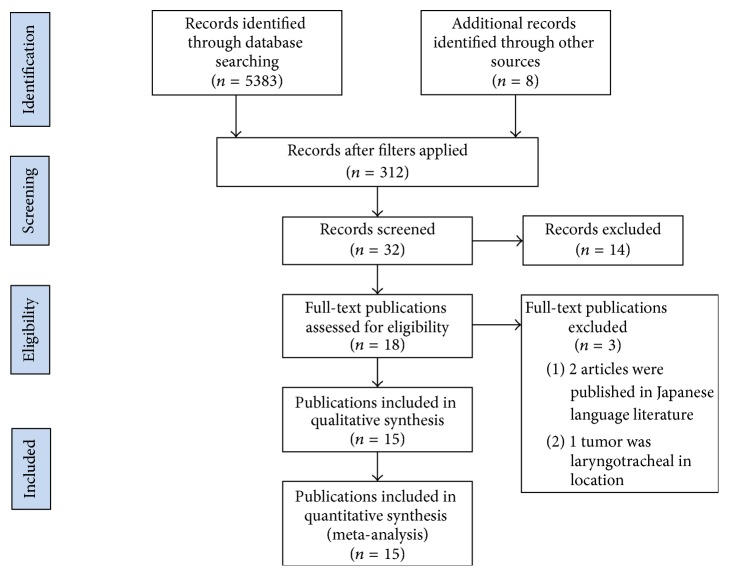
PRISMA diagram of the literature selection process.

A neck computed tomography (CT) scan demonstrated an intraluminal tracheal mass, 2.2 × 2.1 × 1.3 cm in size, obstructing approximately 75% of the tracheal lumen (Figure 1). The tumor origin was 6 cm caudal to the true vocal folds, at the level of the innominate artery. There was a suspicion of extraluminal extension but no calcification appreciated on CT (Figure 1).

Initially, the patient underwent endoscopic debulking of tumor to restore the airway and obtain pathology specimens. Approximately 75% of the gross tumor was removed at that time. Based on the final pathology report from these specimens, the patient was diagnosed with grade 1 chondrosarcoma of the trachea.

Definitive treatment for this patient was open tracheal resection. Transcervical exposure with mini-sternotomy was performed and endotracheal endoscopic video-assisted methylene blue was injected transtracheally to identify the margins of the tracheal resection. Once the tracheal margins were marked and visualized externally, the innominate artery was retracted while a 4 cm tracheal sleeve, including the tumor, was resected. On gross appearance, the tumor was firm, mucosalized, and cartilaginous. Margins were clear by 1.2 cm superiorly and 1.4 cm inferiorly. The free ends of the trachea were approximated by primary anastomosis, using 2-0 and 4-0 polydioxanone sutures, placed externally submucosally in standard fashion. Grillo stitches were placed to remove tension from the trachea in case of excessive patient movement.

The postoperative course was uneventful. The patient remained on a ventilator with the endotracheal tube balloon positioned distal to the tracheal anastomosis for 48 hours. He then underwent flexible bronchoscopy, which confirmed that the mucosa of the anastomosis appeared “sealed.” The patient was then weaned off the ventilator, extubated, and placed on prophylactic proton-pump inhibitor and Valsalva/anticoughing precautions with head of bed elevation. A swallow study the following day was normal, a diet was begun, and the patient was discharged on postoperative day four. The patient was alive without disease recurrence 1 year after surgery, based on follow-up CT (Figure 1) and in office bronchoscopy. In this case, the patient was elected by clinical judgment to be kept intubated for 48 hours, pending future bronchoscopy for mucosal healing before extubation due to the degree of tension placed on the 4 cm sleeve anastomosis intraoperatively and the proximity of the tracheal anastomosis to the innominate artery—clinically anastomotic failure was deemed to be highly morbid in this diabetic, morbidly obese man.

## 4. Discussion

Chondrosarcomas are rare, accounting for only 0.2% of malignancies of the head and neck [21]. These cartilaginous tumors most frequently originate in the pelvis, femur, humerus, and ribs. Only 10–12% of all chondrosarcomas occur in the head and neck area, where the most common sites of origin include the larynx and maxillonasal regions [22, 23]. Our analysis of 16 cases, including the present one, revealed that these tumors occur most often in the elderly, with an average age of 61.3 years at the time of diagnosis and a range of 34–87 years. Two cases occurred in younger males, aged 34 and 35, with Maffucci Syndrome, a rare disease in which diffuse enchondromatosis and cutaneous hemangiomas occur throughout the body [18, 20]. There is a considerable propensity for males, as only one of the recorded patients was female. These tumors occur most frequently in the lower third of the trachea (43.8%), followed by the upper third (37.5%) and the middle third (18.8%). The average tumor size is 3.0 cm, with a range of 2.0–6.5 cm.

Patients with tracheal chondrosarcoma are often asymptomatic until the tumor occludes greater than 75% of the tracheal lumen [14, 17]. The most commonly reported symptoms include dyspnea (the most common symptom, occurring in 100% of these patients), nonproductive cough, and wheeze. As the “alarm sign” of hemoptysis is present only in 12.5% of these cases, the above combination of symptoms can often lead to misdiagnosis of this condition as an acute exacerbation of asthma or chronic obstructive pulmonary disease (COPD) [12, 14, 16, 17].

Computed tomography (CT) is the imaging method of choice for tracheal chondrosarcomas [16]. CT allows for the determination of tumor size, location, presence/absence of calcification, degree of luminal obstruction, and the presence/absence of extratracheal extension [16, 17]. Calcification is present in 71.4% of these tumors and extratracheal extension is observed in 78.6%.

Bronchoscopy is the gold standard for diagnosis, as it allows for collection of pathology specimens as well as for symptomatic treatment of airway compromise via endoscopic resection or laser debulking of the tumor [16].

Unlike chondrosarcomas of the bone and lung, chondrosarcomas of the head and neck tend to be low-grade and slow-growing, with low risk of metastasis [14, 24]. Lymph node involvement occurs in only 5% of cases, and distant metastases occur in only 7–18% [24, 25]. Thus, the lethality of these tumors stems not from distant metastasis but from local aggression, intra-axial invasion, and a high local recurrence rate if not adequately resected [24–27]. Of note, 71% of high-grade (grade 3) chondrosarcomas of the head and neck present with distant metastases [24, 28, 29].

There is one reported case of tracheal chondrosarcoma, which developed distant metastases following malignant transformation of an incompletely resected tracheal chondroma into a grade 3 chondrosarcoma—a case which proved fatal even after multiple resections, 14 years following diagnosis and initial therapy [12].

Definitive treatment for the majority of the patients in this review (11/16) was open surgical resection, with negative margins, of the involved portion of the trachea. No recurrences have been reported in this group of patients, to date. Contrastingly, recurrent disease has been reported in both of the patients (100%) who underwent endoscopic resection as definitive treatment of the tumor [6, 14]. External beam radiotherapy was utilized as adjunctive treatment following laser resection of the tumor in one patient with grade 1 chondrosarcoma in whom open resection was absolutely contraindicated, and the patient remained disease-free 7 years later [19]. Adjuvant radiotherapy has also been used alongside repeat endoscopic resection to treat disease recurrence in a patient who initially underwent endoscopic resection as definitive treatment, after he had refused open resection of the tumor. This patient was alive with the disease at 1-year follow-up [14].  Table 3 shows the extracted, combined patient surveillance status at follow-up and the disease recurrence rate with regard to treatment modality [6–20].

As chondrosarcomas of the trachea are exceedingly rare, a review of other head and neck chondrosarcoma subsites and whole body sites with treatment analysis should also be considered when discussing treatment options. In the head and neck, one of the most common subsites for chondrosarcomas to originate is the larynx. Achieving adequate resection of these tumors while sparing laryngeal function is ideal. Endoscopic removal, thyrotomy, laryngofissure, are partial laryngectomy are laryngeal function-sparing techniques that can achieve wide excision of the tumor along with a sufficient margin of uninvolved cartilage [30]. Unfortunately, however, up to 75% of these tumors involve the cricoid cartilage, which is considered crucial for laryngeal function [30–32]. Traditionally, total laryngectomy has been recommended for tumors involved in greater than 50% of the cricoid cartilage. However, CO2 lasering and reconstructive techniques such as thyrotracheal anastomosis over a stent and rib graphs suggest that laryngeal function can still be preserved in these patients [30, 33]. Importantly, recurrence rates of laryngeal chondrosarcomas have been reported at 35–40%, with a tendency for late recurrence [30]. Thus, patients with head and neck chondrosarcomas should be followed continuously, with a high level of suspicion for recurrence [22]. The prognosis of low-grade laryngeal chondrosarcoma, when treated adequately, shows an overall survival rate of 95% at 10 years. Tumor grade, composition, and type of surgical procedure performed should be considered when determining individual prognoses [30, 33–35].

As there are no evidence-based recommendations for margin control in head and neck chondrosarcomas, broadening our discussion to include the optimal treatment for whole body chondrosarcomas warrants consideration. Recent studies of low-grade chondrosarcomas in the extremities have found that aggressive intralesional curettage, with or without cryosurgery at the margin, may be an acceptable alternative to wide excision of these lesions [36, 37]. These initial studies demonstrate that recurrence rates after aggressive intralesional excision are relatively low and that they do not differ, significantly, from those of wide excision (3.8% recurred in intralesional excision and 1.8% recurred in wide excision). Postsurgical functionality is greater in patients treated conservatively with intralesional curettage than in those treated aggressively with wide excision [22, 35]. These findings may suggest that the aggressiveness of treatment modality of low-grade chondrosarcomas of the extremities may be tailored for each individual based on tumor characteristics that may help predict tumor behavior, such as location and degree of differentiation. For tumors originating in the trachea, recurrences may be more difficult to treat and aggressive intralesional treatment is not a likely viable option, as the integrity of the tracheal wall must be maintained in order to keep the air filled luminal space separate from the negative pressure mediastinal cavity. Of note, this particular long bone surgical concept difficultly reconciles with the data collected from the tracheal patients examined in this review because, in this study, the mean tumor size was 3.0 cm (range, 2.0–6.5 cm) and extratracheal extension was present in 78.6% of these patients. Partial excision, or complete gross tumor endoscopic resection with adjunctive treatment to the margin, is likely not feasible in most cases of tracheal chondrosarcoma, due to their size at diagnosis and/or the presence of extratracheal extension compromising the integrity of the tracheal wall. What this evolving long bone treatment paradigm may help show is that low-grade chondrosarcomas likely do not require the same degree of margin control as do other sarcomas in minimizing local recurrence rates. From this literature and the laryngeal chondrosarcoma literature, it seems evident that less aggressive margin clearance into normal tissue applies in low-grade chondrosarcomas, achieving acceptably low recurrence rates—unlike most other sarcomas. However, observation and recurrence in the trachea are not the same as an extremity, as the trachea must retain its patency for air-flow and tumors of any appreciable size would become symptomatic. Further research is needed on the overall recurrence rates of chondrosarcomas of varying histologic grades with regard to the precise degree of margin control.

Traditionally, chondrosarcomas have been thought to be relatively radioresistant, due, in part, to their low-grade nature [16, 24]. However, in recent studies, head and neck chondrosarcomas have shown a response to radiotherapy in certain circumstances [19, 24, 38–40]. As such, radiotherapy is now utilized as adjuvant therapy in high-grade tumors to combat local recurrence after surgical excision, inoperable tumors for palliation, recurrent tumors, patients with incomplete resection or positive margins, patients who are poor surgical candidates, and patients who refuse margin-clearing surgery [16, 24, 30].

Currently, no evidence exists to support the use of chemotherapy in the treatment of chondrosarcomas, although it is used by some in the treatment of high-grade tumors with a greater risk for metastases and should be considered in those with lymph node metastasis [22, 41].

## Figures and Tables

**Table 1:** Selected characteristics of 18 cases of tracheal chondrosarcoma.

Year	First author	Sex	Age	Symptoms	Symptom duration (months)	Location	Grade (1–3)	Extratracheal extension	Size (cm)	Calcification	Treatment	Number of recurrences	Follow-up (years)
1967	Daniels	M	73	D, C, W	7	Lower	2	NR	2.5	NR	(1) ER^‡^	5 (local)	AWD, 3.0
											(2) ER		
1973	Fallahnejad	F	48	D, C, W	16	Upper	NR	Present	4.0	None	R	0	NED, 5.0
1978	Weber	M	71	D, C, Ho	4	Middle	NR	Present	3.0	Present	R	0	NED, 5.0
1985	Slasky	M	58	D	24	Lower	2	Present	2.0	Present	R	0	NED, 2.5
1986	Arévalo	M	74	D, P	0.25	Upper	1	Present	2.0	Present	R	0	NED, 1.0
1988	Matsuo	M	72	D, He	7	Middle	1	Present	5.0	None	(1) IR^‡^	1	NED, 0.5
											(2) LaR		
											(3) R		
1990	Salminen	M	57	D, C	3	Lower	3	Present	2.3	None	IR	5 (local & distant)	DOD, 14
1994	Leach	M	72	D	36	Lower	3	Present	6.5	Present	R	NR	NR
1998	Farrell	M	87	D	12	Middle	2	Present	3.0	NR	(1) ER^*∗*,‡^	2 (local)	AWD, 1.0
											(2) ER		
											(3) Rad		
1997	Kiriyama	M	54	D, W	1	Lower	1	NR	2.0	Present	LaDeb/R^§^	0	NED, 3.5
2003	Maish	M	78	D	3	Lower	1	Present	NR	None	LaDeb/R^§^	0	NED, 0.5
2008	Umezu	M	34	D, He, W	8	Upper	1	Present	2.5	Present	R	0	NED, 6.3
2009^†^	Wagnetz	M	34	D, C, W	18	Upper	2	None	2.0	Present	ER/R^§^	NR	NR
2010	Mendonça	M	72	D, W, O	12	Upper	1	Present	NR	Present	LaDeb/Rad^§^	0	NED, 7.0
2010^†^	de Almeida	M	35	D	NR	Upper	2	None	NR	Present	R	NR	NR
2016	Kutzner	M	61	D	2	Lower	1	None	2.2	Present	ER/R^§^	0	NED, 1.0

M, male; F, female; NR, not recorded; C, cough; D, dyspnea; W, wheeze; Ho, hoarse; P, pneumonia; He, hemoptysis; O, odynophagia; R, resection; ER, endoscopic resection; IR, incomplete resection; LaR, laser resection; LaDeb/R, laser debulking/resection; UNK, unknown; Rad, radiotherapy; NED, no evidence of disease; AWD, alive with disease; DOD, died of disease. ^*∗*^Refused resection. ^†^Patient with Maffucci Syndrome. ^‡^Patient with recurrences treated in numerical order. ^§^Patient symptomatically treated with laser debulking or with endoscopic resection prior to definitive treatment of resection or radiotherapy.

**Table 2:** Analysis of selected characteristics of tracheal chondrosarcoma [6–20].

Characteristic		Mean	Median	Mode	Range
Age (years)		61.3	66	72	34–87
Size (cm)		3.0	2.5	2.0	2.0–6.5
Symptom duration (months)		10.2	7	7	0.25–36

	Number of cases/number reported	Percentage			

Male	15/16	93.8%			
Symptoms					
Dyspnea	16/16	100.0%			
Wheeze	6/16	37.5%			
Cough	5/16	31.3%			
Hemoptysis	2/16	12.5%			
Hoarseness	1/16	6.3%			
Odynophagia	1/16	6.3%			
Grade					
1	7/14	50.0%			
2	5/14	35.7%			
3	2/14	14.3%			
Tracheal location					
Lower	7/16	43.8%			
Upper	6/16	37.5%			
Middle	3/16	18.8%			
Calcification	10/14	71.4%			
Extratracheal	11/14	78.6%			

**Table 3:** Patient recurrence rates with regard to treatment modality [6–20].

Definitive treatment modality	Number of cases					Length of follow-up (years)			Recurrence rate
	Follow-up reported	REC	NED	AWD	DOD	Mean	Median	Range	
Open resection ([7–10, 13, 15–18, 20], present case)	11	0	10	0	0	3.1	3	0.5–6.3	0%
Endoscopic resection only [6, 14]	2	7	0	2	0	2	2	1–3	100%
Incomplete resection [11, 12]	2	5	1	0	1	7.3	7.3	0.5–14.0	100%

Endoscopic resection + XRT [19]	1	0	1	0	0	7	7	7	0%

Overall	16	12^*∗*^	13	2	1	4.8	5	0.5–14.0	75%

XRT: external beam radiation therapy; REC, recurrence; NED, no evidence of disease; AWD, alive with disease; DOD, died of disease. ^*∗*^Multiple recurrences in small number of patients.
